# Effect of CRISPR Knockout of *AXIN1* or *ARID1A* on Proliferation and Migration of Porcine Hepatocellular Carcinoma

**DOI:** 10.3389/fonc.2022.904031

**Published:** 2022-05-20

**Authors:** Lobna Elkhadragy, Kimia Dasteh Goli, William M. Totura, Maximillian J. Carlino, Maureen R. Regan, Grace Guzman, Lawrence B. Schook, Ron C. Gaba, Kyle M. Schachtschneider

**Affiliations:** ^1^ Department of Radiology, University of Illinois at Chicago, Chicago, IL, United States; ^2^ Department of Biochemistry and Molecular Genetics, University of Illinois at Chicago, Chicago, IL, United States; ^3^ Department of Pathology, University of Illinois at Chicago, Chicago, IL, United States; ^4^ Department of Animal Sciences, University of Illinois at Urbana-Champaign, Urbana, IL, United States; ^5^ National Center for Supercomputing Applications, University of Illinois at Urbana-Champaign, Urbana, IL, United States

**Keywords:** liver cancer, hepatocellular carcinoma, porcine models, ARID1A, AXIN1, CRISPR, gene knockout

## Abstract

Hepatocellular carcinoma (HCC) is an aggressive disease lacking effective treatment. Animal models of HCC are necessary for preclinical evaluation of the safety and efficacy of novel therapeutics. Large animal models of HCC allow testing image-guided locoregional therapies, which are widely used in the management of HCC. Models with precise tumor mutations mimicking human HCC provide valuable tools for testing precision medicine. *AXIN1* and *ARID1A* are two of the most frequently mutated genes in human HCC. Here, we investigated the effects of knockout of *AXIN1* and/or *ARID1A* on proliferation, migration, and chemotherapeutic susceptibility of porcine HCC cells and we developed subcutaneous tumors harboring these mutations in pigs. Gene knockout was achieved by CRISPR/Cas9 and was validated by Next Generation Sequencing. *AXIN1* knockout increased the migration of porcine HCC cells but did not alter the cell proliferation. Knockout of *ARID1A* increased both the proliferation and migration of porcine HCC cells. Simultaneous knockout of *AXIN1* and *ARID1A* increased the migration, but did not alter the proliferation of porcine HCC cells. The effect of gene knockout on the response of porcine HCC cells to two of the most commonly used systemic and locoregional HCC treatments was investigated; sorafenib and doxorubicin, respectively. Knockout of *AXIN1* and/or *ARID1A* did not alter the susceptibility of porcine HCC cells to sorafenib or doxorubicin. Autologous injection of CRISPR edited HCC cells resulted in development of subcutaneous tumors in pigs, which harbored the anticipated edits in *AXIN1* and/or *ARID1A*. This study elucidates the effects of CRISPR-mediated knockout of HCC-associated genes in porcine HCC cells, and lays the foundation for development and utilization of genetically-tailored porcine HCC models for *in vivo* testing of novel therapeutic approaches in a clinically-relevant large animal model.

## Introduction

Primary liver cancer is the sixth most commonly diagnosed cancer and the third leading cause of cancer death worldwide (1). The predominant type of primary liver cancer is hepatocellular carcinoma (HCC), comprising 75-85% of cases (1). HCC is an aggressive malignancy with poor prognosis and an estimated 5-year survival rate of 18% (2). Currently available therapies provide only a modest survival benefit to HCC patients (3), and there is a critical need to develop novel therapeutic approaches. As HCC treatments move toward precision approaches based on tumor genetic and molecular alterations (4–7), elucidating the role of frequently occurring gene alterations on tumor progression and therapeutic susceptibility is essential.

Deep sequencing analysis of hundreds of human HCC tumors and liquid biopsies has revealed a wealth of knowledge about the genomic landscape of HCC (8–12). Mutations in axis inhibition protein 1 (*AXIN1*) and AT-rich interaction domain 1A (*ARID1A*) are among the most frequent genetic alterations in human HCC (9, 10). *AXIN1*, a negative regulator of the Wnt pathway, is mutated in 8-10% of HCC. Most of these mutations are loss-of-function mutations (13). *ARID1A* is a subunit of the SWI/SNF chromatin-remodeling complex. It is mutated in 10-15% of HCCs as well as in several types of cancer (14, 15). Most *ARID1A* mutations in tumors are inactivating frameshift or nonsense mutations that exist throughout the gene and result in loss of protein expression (15).

Development of novel therapeutic strategies necessitates the availability of animal models that recapitulate the phenotype and genotype of the human disease for preclinical testing. As locoregional therapies are widely employed in the management of HCC (3, 4), the availability of large animal models that support testing such treatment modalities is essential. Large animal models allow testing image-guided therapies, such as transarterial chemoembolization, intra-arterial local immunotherapy, and intra-arterial tumor-targeting drug carriers, that are not feasible to test in mouse models due to their small size (16). HCC models have been recently developed in pigs by orthotopic implantation or chemical induction (16–20). Our group has developed the implantation model using transgenic Oncopigs, which harbor inducible *TP53^R167H^* and *KRAS^G12D^* transgenes (17, 21, 22). This model recapitulates cytologic, transcriptional, and histologic features of human HCC (21), and has been used for testing locoregional therapies using human-scale tools (16).

Investigating the role of clinically relevant gene mutations in porcine HCC cells is a necessary step that leads to effective utilization of porcine HCC models for testing novel precision medicine approaches, including systemic and locoregional therapies. The objective of the current study was to investigate the effects of *AXIN1* and/or *ARID1A* mutations on proliferation, migration, and therapeutic susceptibility of porcine HCC cells. Porcine HCC cells developed from Oncopigs were used in the study, as they recapitulate human HCC characteristics (17, 21). By employing clustered regularly interspaced short palindromic repeats (CRISPR)/Cas9 gene editing, loss-of-function mutations similar to those occurring in human HCC were induced in porcine HCC cells, and the effects of gene knockout on HCC cell phenotype and treatment response was investigated.

## Materials and Methods

### Animals

All animal procedures were approved by The University of Illinois Institutional Animal Care and Use Committee (IACUC). Oncopigs heterozygous for the transgene construct were utilized in this study. In total, 4 Oncopigs (A272, A273, A274, and A343) were used in this study.

### Cell Culture

Porcine HCC cells were developed from Oncopigs by collagenase digestion of a surgically resected liver section, followed by induction of transgene expression in isolated hepatocytes using Cre recombinase, as previously described (17, 23). Porcine HCC cells were maintained in DMEM supplemented with 10% FBS and 1% Penicillin-Streptomycin. All culture media and supplements were purchased from Gibco/Thermo Fisher Scientific, Waltham, MA.

### CRISPR/Cas9 Gene Editing

gRNAs were designed using CRISPOR (www.crispor.tefor.net) as previously described (23, 24) and the AltR^®^ CRISPR/Cas9 system (IDT Corporation, IL, USA) was used for gene editing. Each gRNA was synthesized by incubating equimolar ratios of crRNA (sequences in **Table 1**, synthesized by IDT Corporation) and tracrRNA (#1075927; IDT Corporation) at 95°C for 5 minutes followed by cooling to room temperature. Purified *S. pyogenes* Cas9 nuclease (#1081058; IDT Corporation) diluted in Opti-MEM (#31985062; Gibco/Thermo Fisher Scientific) was then combined with the gRNA at equimolar ratio to form a ribonucleoprotein (RNP) complex. Porcine HCC cells were transfected with 25 nM RNP using Lipofectamine CRISPRMAX transfection reagent (#CMAX00003; Invitrogen, Carlsbad, CA) following the manufacturer’s instructions.

**Table 1:** Oligonucleotides used in the study.

crRNAs	
***AXIN1 crRNA#1***	5’- GGCCCACTTCAAGTACGGCG -3’
***AXIN1 crRNA#2***	5’- CCCGTCCTGATCGTCGAGCA -3’
***AXIN1 crRNA#3***	5’- GCACTCCCTGCTCGACGATC -3’
***ARID1A crRNA***	5’- GGACTTTGCTGGTTGTAATA-3’
**PCR primers for targeted sequencing**	
**AXIN1-For**	5’-ACACTCACGACATGGTTCTACACACAGCTTCTGCTCTGGGAA-3’
**AXIN1-Rev**	5’-TACGGTAGCAGAGACTTGGTCTCTCCTCGTTCGAGTCGCAG-3’
**ARID1A-For**	5’-ACACTCACGACATGGTTCTACATAAACTACCAGAAGTATCAGTGCT-3’
**ARID1A-Rev**	5’-TACGGTAGCAGAGACTTGGTCTTGGCTGCTGGGAATATGGAG-3’

The shaded regions represent adaptor sequences for barcode attachment.

### Analysis of Gene Editing by Next-Generation Sequencing (NGS)

Genomic DNA was extracted from porcine HCC cells using QuickExtract DNA Extraction Solution (#QE09050; Lucigen, Middleton, WI) as previously described (23). The genomic locus that flanks the Cas9 target site was amplified by PCR for 28 cycles using primers listed in **Table 1**. These primers contain NGS adaptors used to add additional adaptor sequences and barcodes as part of the Fluidigm library preparation. PCR products were then provided to the UIC Genome Research Core for library preparation and sequencing. Briefly, a second PCR was done to attach Fluidigm barcode and NGS adaptor sequences to the amplicons generated. Samples were then pooled and targeted NGS was performed using a MiSeq instrument (2 x150 kit, Illumina, San Diego, CA) following the manufacturer’s instructions. The gene editing efficiency was calculated using CRISPRESSO2 alignment tool (crispresso.pinellolab.partners.org) (25), which quantifies the frequency of sequences containing indels.

### Isolation of Single Cell Clones

Two days post-RNP transfection, the cells were diluted to 1 cell/100 µl medium and single cells were seeded into each well of a 96-well plate. Visual inspection was done to confirm the presence of a single cell/colony per well. When a colony reached about 80% confluency, cells were trypsinized and re-seeded into two wells of another 96-well plate. One well was used for DNA extraction using 20 µl QuickExtract DNA Extraction solution and analyzed by NGS as described above, and the other well was expanded.

### Western Blotting

2.5 x 10^6^ cells were seeded in 6-well plates and kept in a cell culture incubator at 37°C and 5% CO_2_ overnight. Next day, the cells were washed with ice-cold PBS and lysed with 250 µl RIPA buffer (#AAJ63306AK; Thermo Fisher Scientific) mixed with Halt Protease Inhibitor Cocktail (#78429; Thermo Fisher Scientific). SDS-PAGE using 7.5% polyacrylamide gel was followed by transferring the proteins onto nitrocellulose membranes, and blocking the membranes with 5% non-fat milk in PBS with tween 20 for 30 minutes. Incubation with the primary antibodies was done overnight at 4°C followed by 1 hour incubation with the appropriate secondary antibodies at room temperature. The following primary antibodies were used: anti-AXIN1 (#A0481; Sigma-Aldrich), anti-β-actin (#C4; Santa Cruz technologies), and the following secondary antibodies were used: anti-mouse (#170-6516; Biorad, Hercules, CA) and anti-rabbit (#170-6515; Biorad). The Western blots were visualized by chemiluminescence (#32109; Thermo Fisher Scientific). Band intensities were quantified by ImageJ software.

### Cell Proliferation Assay

Cell proliferation was determined using the CellTiter 96^®^ AQueous One Solution Cell Proliferation Assay Kit (#G3580; Promega, Madison WI) following the manufacturer’s instructions using a BioTek 800 TS Absorbance Reader (BioTek, Winooski, VT).

### Cell Migration Assay

Cell migration was analyzed using a modified two chamber transwell system (#353097; Corning, Corning, NY, USA). Cells were detached by trypsin/EDTA, washed once with serum-free medium, and then resuspended in serum-free medium. Complete culture medium with 10% FBS was added to each bottom well. In total, 25,000 cells were added in each transwell insert and allowed to migrate for 16-18 hours in a 37°C cell incubator, then the cells in the upper surface of the transwell were removed using cotton swabs. The migrated cells attached on the undersurface were fixed with 4% paraformaldehyde for 15 min and stained with crystal violet solution (0.5% in water) for 10 min. Migrated cells were then photographed and counted. The quantitated migration ability was presented as the number of migrated cells per field.

### Chemotherapeutic Susceptibility

The sensitivity of porcine HCC cells to sorafenib and doxorubicin was determined by a dose-response assay. Briefly, 7 x 10^3^ cells/well were seeded in 96-well plates. The following day, culture medium was replaced with fresh medium containing serial dilutions of doxorubicin (#1208; Selleck Chemicals, Houston, TX) or sorafenib (#6814; Tocris, Bristol, UK). Cell viability was assessed after 48 hours using an MTS assay (Promega) following the manufacturer’s recommendations.

### Autologous Tumorigenesis Assay

1 x 10^7^ porcine HCC cells were washed twice with PBS, re-suspended in 100 μl PBS, and autologously injected subcutaneously in the flank of an anesthetized pig using a 21 G needle. Oncopig A273 received two injections; control unedited HCC cells at one site and edited HCC cell pool with *ARID1A* edits into another site. Oncopig A343 received injections at 4 distinct sites: unedited cells in two sites, and CRISPR-edited single-cell clones in two sites. These were *AXIN1* ^KO^ cells and cells with *ARID1A* ^KO^ and monoallelic *AXIN1* ^KO^. Injection sites were monitored visually and by palpation. The masses in A273 Oncopig were excised on day 11 post-injection. Biopsies were collected from the subcutaneous masses in A343 Oncopig on day 8 post-injection under ultrasound guidance.

### Histologic Processing

Subcutaneous mass samples were processed for hematoxylin and eosin (H&E) staining and immunohistochemistry (IHC) staining of Arginase-1 using anti-Arginase antibody (#ab91279; Abcam, Cambridge, UK). Whole slides were scanned using a Hamamatsu Nanozoomer scanner (Hamamatsu Photonics, Hamamatsu City Japan), and digital images were visualized with Aperio ImageScope software (Leica, Lincolnshire, IL). Histopathological analyses were performed by a board-certified human pathologist with subspecialty training in Liver and Transplantation Pathology.

### Statistical Analysis

Data were expressed as mean ± standard deviation in proliferation assays and chemotherapeutic susceptibility assays, and as mean ± standard error (S.E.) in migration assays. Half-maximal inhibitory concentration (IC_50_) values were determined by non-linear regression analysis using GraphPad Prism 8 (GraphPad, San Diego, CA) from plots of relative percent viability versus log_10_ drug concentration. All assays were repeated three times and a representative figure is presented. Statistical significance was determined by Student’s *t* test in migration assays or by two-way analysis of variance (ANOVA) in cell proliferation assays, and a *p*-value of less than 0.05 was considered statistically significant.

## Results

### Development and Functional Characterization of *AXIN1* ^KO^ Porcine HCC Cell Lines

**Figure 1:**
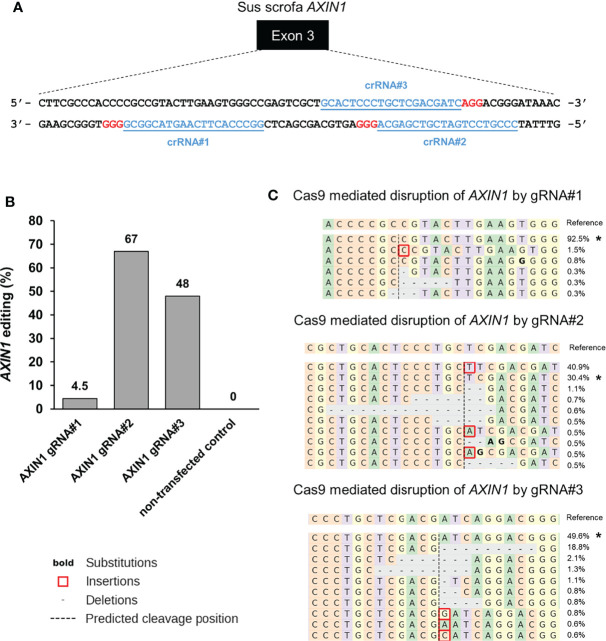
CRISPR/Cas9 disruption of porcine *AXIN1* gene. **(A)** Schematic representation of porcine *AXIN1* locus showing the location of spacer sequences crRNA#1, crRNA#2, and crRNA#3 (underlined blue font). Protospacer adjacent motif (PAM) sequences are marked in red. **(B)** Comparing CRISPR/Cas9 editing efficiency of three individual *AXIN1* targeting gRNAs. Porcine A272 HCC cells were transfected with ribonucleoproteins (RNPs) comprising Cas9 and each gRNA. Non-transfected cells were used as control. Genomic DNA was collected two days post-transfection and analyzed by targeted NGS. The bar graph depicts the percentages (%) of total reads that displayed indels at the gRNA target site occurring as a result of non-homologous end joining (NHEJ). **(C)** Type and frequency of *AXIN1* indels detected by targeted NGS analysis mapped to the reference sequence. The percentage of reads for each sequence are shown on the right. The asterisk (*) indicates/marks non-edited reads. The top 10 reads are shown for cells transfected with gRNA#2 or gRNA#3. Dashed line, predicted Cas9 cleavage position; red box, insertion; dash, deleted base.

To generate loss-of-function mutations in *AXIN1* similar to those occurring in human HCC, three individual gRNAs were designed and screened in porcine HCC cells (**Figure 1A**). The gene editing efficiency in cells transfected with Cas9 complexed with gRNA#1, gRNA#2, and gRNA#3 was 4.5%, 67%, and 48%, respectively (**Figure 1B**). As gRNA#2 resulted in the highest editing efficiency among the three tested gRNAs, it was used for all subsequent *AXIN1* disruption experiments. Consistent with the anticipated CRISPR/Cas9 effect, small insertions and deletions (indels) occurred around the predicted Cas9 cleavage position (**Figure 1C**). These results demonstrate successful CRISPR/Cas9 disruption of porcine *AXIN1* gene.

**Figure 2:**
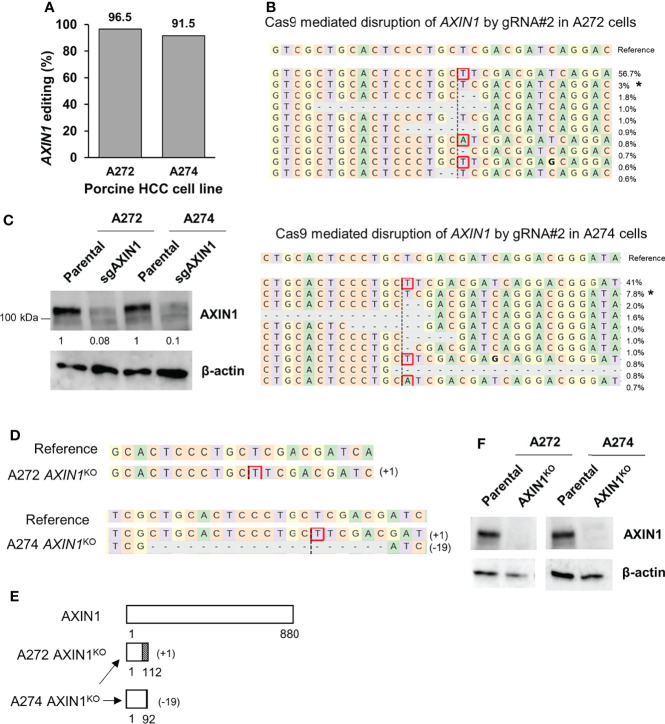
Generation of *AXIN1* ^KO^ porcine HCC cells by CRISPR/Cas9. **(A)** CRISPR/Cas9-induced editing of *AXIN1* in A272 and A274 porcine HCC cells using *AXIN1* gRNA#2. Genomic DNA was collected two days post-transfection and analyzed by targeted NGS. The bar graph depicts the percentages (%) of total reads that displayed indels at the gRNA target site. **(B)** Top 10 reads detected by targeted NGS analysis in the two porcine HCC lines mapped to the reference sequence. The percentage of reads of each sequence are shown on the right. The asterisk (*) indicates/marks non-edited reads. Dashed line, predicted Cas9 cleavage position; red box, insertion; dash, deleted base. **(C)** Confirmation of AXIN1 protein depletion by Western blotting in A272 and A274 porcine HCC cell pools transfected with Cas9 and gRNA#2. The cells were lysed two days post-transfection and analyzed by Western blotting using an anti-AXIN1 antibody. β-actin was used as a loading control. **(D–F)** Analysis of *AXIN1* ^KO^ single cell clones isolated from A272 and A274 porcine HCC cells transfected with Cas9 and *AXIN1* gRNA#2. **(D)** Reads detected by targeted NGS analysis mapped to the reference sequence (top) for A272 and A274 *AXIN1* ^KO^ cells. Dashed line, predicted Cas9 cleavage position; red box, insertion; dash, deleted base. **(E)** Schematic representation of predicted translation of AXIN1 protein in the isolated *AXIN1* ^KO^ HCC cells. The dotted region represents amino acids with frameshift mutation. **(F)** Confirmation of complete loss of AXIN1 protein in A272 *AXIN1* ^KO^ and A274 *AXIN1* ^KO^ cells by Western blotting.

Next, we aimed to isolate *AXIN1* knockout (KO) cells for functional characterization of the effects of AXIN1 loss. Two porcine HCC cells (A272 and A274) were transfected with *AXIN1* gRNA#2 complexed with Cas9, resulting in 96.5% and 91.5% *AXIN1* editing, respectively (**Figures 2A, B**). Consistent with NGS results, Western blotting demonstrated greater than 90% depletion of AXIN1 protein in both these cell pools (**Figure 2C**). Single cell clones were isolated and screened for *AXIN1* KO mutations by NGS analysis. A272 and A274 *AXIN1* ^KO^ clones with mutations causing frameshift and premature stop codons were selected (**Figure 2D**). These clones had a predicted protein length nearly 1/8^th^ the size of AXIN1 (**Figure 2E**). Complete AXIN1 protein loss was confirmed in these two clones by Western blotting (**Figure 2F**).

**Figure 3:**
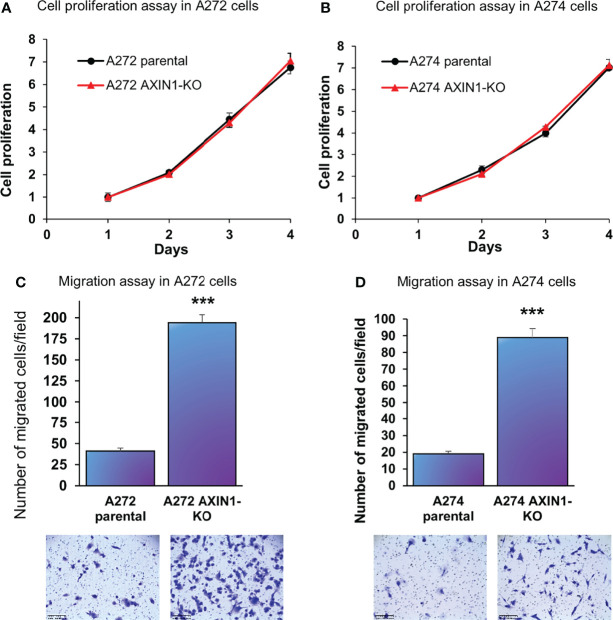
*AXIN1* knockout increases the migration, but does not alter the proliferation, of porcine HCC cells. **(A, B)** Cell proliferation was determined for A272 **(A)** and A274 **(B)** parental and *AXIN1* ^KO^ cells by MTS assay. Cell viability at different time points (days) was measured and expressed as *A*_490_ normalized to values of day 1. Statistical analysis was conducted to compare viability of the different cell lines at each time point by two-way ANOVA. No significant difference was detected between the cell lines at p < 0.05. **(C, D)** Migration of A272 *AXIN1* ^KO^ **(C)** and A274 *AXIN1* ^KO^ **(D)** cells in comparison to parental cells was assessed by transwell cell migration assay. Quantitated migration ability is presented as the number of migrated cells per field. Values in the bar graph represent mean ± S.E. (n = 6 fields). ***, p < 0.0001. Representative images of migrated cells stained with crystal violet are shown below each bar graph. Scale bar, 250 μm.

In both A272 and A274 porcine HCC cells, KO of *AXIN1* did not change the rate of cell proliferation measured by MTS assays (**Figures 3A, B**). However, *AXIN1* ^KO^ cells exhibited a significantly higher migration as compared with parental cells in trans-well migration assays (**Figures 3C, D**).

### Development and Functional Characterization of *ARID1A* ^KO^ Porcine HCC Cell Lines

**Figure 4:**
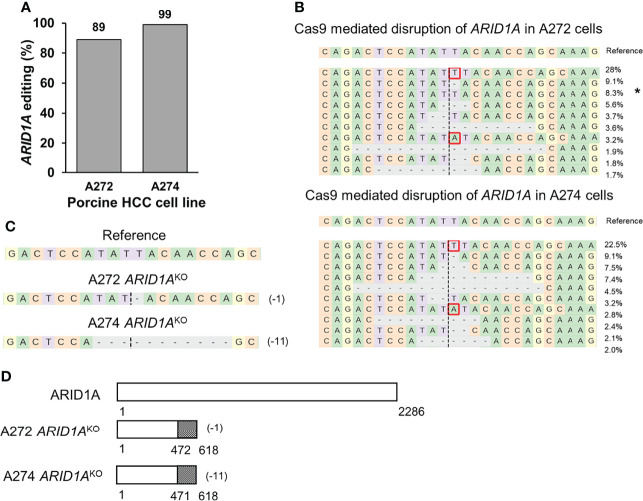
Generation of *ARID1A* ^KO^ porcine HCC cells by CRISPR/Cas9. **(A)** CRISPR/Cas9-induced editing of *ARID1A* in A272 and A274 porcine HCC cells. Genomic DNA was collected two days post-transfection and analyzed by targeted NGS. The bar graph depicts the percentages (%) of total reads that displayed indels at the gRNA target site. **(B)** Top 10 reads detected by targeted NGS analysis in the two porcine HCC lines mapped to the reference sequence. The percentage of reads of each sequence are shown on the right. The asterisk (*) indicates/marks non-edited reads. Dashed line, predicted Cas9 cleavage position; red box, insertion; dash, deleted base. **(C, D)** Analysis of *ARID1A* ^KO^ single cell clones isolated from A272 and A274 porcine HCC cells transfected with Cas9 and *ARID1A* gRNA. **(C)** Reads detected by targeted NGS analysis mapped to the reference sequence for A272 and A274 *ARID1A* ^KO^ cells. Dashed line, predicted Cas9 cleavage position; red box, insertion; dash, deleted base. **(D)** Schematic representation of predicted translation of ARID1A protein in the isolated *ARID1A* ^KO^ HCC cells. The dotted region represents amino acids with frameshift mutation.

A previously validated gRNA was used to target porcine *ARID1A* gene (23). Transfection of A272 and A274 porcine HCC cells with Cas9 complexed with *ARID1A* gRNA resulted in 89% and 99% editing, respectively, at the expected target region as demonstrated by NGS (**Figures 4A, B**). Single cell clones were isolated from these cells and screened for *ARID1A* KO mutations. A272 and A274 *ARID1A* ^KO^ clones had deletion of a single nucleotide, or 11 nucleotides, respectively, predicted to result in translation of a truncated ARID1A protein (**Figures 4C, D**). Confirmation of protein loss was not feasible due to lack of availability of an antibody that targets porcine ARID1A protein (23).

**Figure 5:**
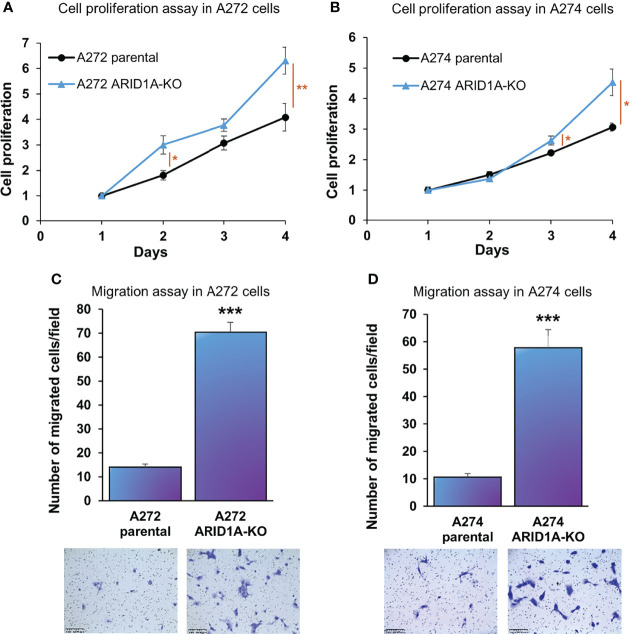
*ARID1A* knockout increases the proliferation and migration of porcine HCC cells. **(A, B)** Cell proliferation was determined for A272 **(A)** and A274 **(B)** parental and *ARID1A* ^KO^ cells by MTS assay. Cell viability at different time points (days) was measured and expressed as *A*_490_ normalized to values of day 1. Statistical analysis was conducted to compare viability of the different cell lines at each time point by two-way ANOVA. *, p < 0.05; **, p < 0.005. **(C, D)** Migration of A272 *ARID1A* ^KO^ **(C)** and A274 *ARID1A* ^KO^ **(D)** cells in comparison to parental cells was assessed by transwell cell migration assay. Quantitated migration ability is presented as the number of migrated cells per field. Values in the bar graph represent mean ± S.E. (n = 6 fields). ***, p < 0.0001. Representative images of migrated cells stained with crystal violet are shown below each bar graph. Scale bar, 250 μm.

*ARID1A* KO resulted in a significant increase in proliferation of both A272 and A274 porcine HCC cells (**Figures 5A, B**). Also, *ARID1A* KO increased the migration of porcine HCC cells as compared with parental cells (**Figures 5C, D**).

### Simultaneous KO of *AXIN1* and *ARID1A* in Porcine HCC Cells

**Figure 6:**
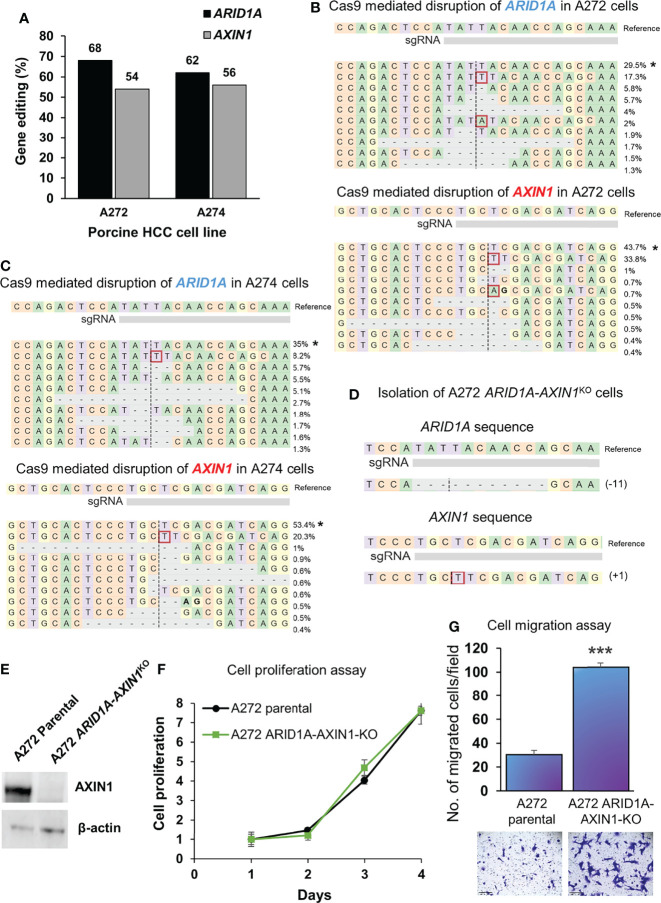
Simultaneous knockout of *ARID1A* and *AXIN1* in Oncopig HCC cells increases the migration, but does not alter the proliferation of porcine HCC cells. **(A)** CRISPR/Cas9-induced editing of *AXIN1* and *ARID1A* in A272 and A274 porcine HCC cells using *AXIN1* gRNA#2 and *ARID1A* gRNA. Genomic DNA was collected two days post-transfection and analyzed by targeted NGS. The bar graph depicts the percentages (%) of total reads that displayed indels at the gRNA target site. **(B, C)** Top 10 reads detected by targeted NGS analysis in the two porcine HCC lines mapped to the reference sequence. The percentage of reads of each sequence are shown on the right. The asterisk (*) indicates/marks non-edited reads.. Dashed line, predicted Cas9 cleavage position; red box, insertion; dash, deleted base. **(D)** NGS Analysis of *ARID1A-AXIN1* ^KO^ single cell clone isolated from A272 porcine HCC cells transfected with Cas9, *AXIN1* gRNA#2, and *ARID1A* gRNA. Dashed line, predicted Cas9 cleavage position; red box, insertion; dash, deleted base. **(E)** Confirmation of complete loss of AXIN1 protein in A272 *ARID1A-AXIN1* ^KO^ porcine HCC cells by Western blotting. The cells were lysed two days post-transfection and analyzed using an anti-AXIN1 antibody. β-actin was used as a loading control. **(F)** Cell proliferation was determined for A272 parental and *ARID1A-AXIN1* ^KO^ cells by MTS assay. Cell viability at different time points (days) was measured and expressed as *A*_490_ normalized to values of day 1. Statistical analysis was conducted to compare viability of the different cell lines at each time point by two-way ANOVA. No significant difference was detected between the cell lines at p < 0.05. **(G)** Migration of A272 *ARID1A-AXIN1* ^KO^ cells in comparison to parental cells was assessed by transwell cell migration assay. Quantitated migration ability is presented as the number of migrated cells per field. Values in the bar graph represent mean ± S.E. (n = 6 fields). ***, p < 0.0001. Representative images of migrated cells stained with crystal violet are shown below each bar graph. Scale bar, 250 μm.

A272 and A274 porcine HCC cells were transfected simultaneously with Cas9 complexed with *AXIN1* gRNA#2 and Cas9 complexed with *ARID1A* gRNA. CRISPR edits were detected in both genes, as determined by NGS. A272 porcine HCC cells had 68% editing in *ARID1A* and 54% editing in *AXIN1*. A274 cells had 62% editing in *ARID1A* and 56% editing in *AXIN1* (**Figures 6A–C**). Single cell clones were isolated from A272 cells transfected with *ARID1A* and *AXIN1* gRNAs. KO of both genes in A272 *ARID1A-AXIN1* ^KO^ clone was confirmed by NGS (**Figure 6D**) and loss of AXIN1 protein was confirmed by Western blotting (**Figure 6E**). A272 *ARID1A-AXIN1* ^KO^ cells proliferated at a similar rate as the parental cells (**Figure 6F**), but showed a significant increase in migration as compared with parental cells (**Figure 6G**).

### *AXIN1* and/or *ARID1A* KO Does Not Alter the Susceptibility of Porcine HCC Cells to Sorafenib or Doxorubicin

**Figure 7:**
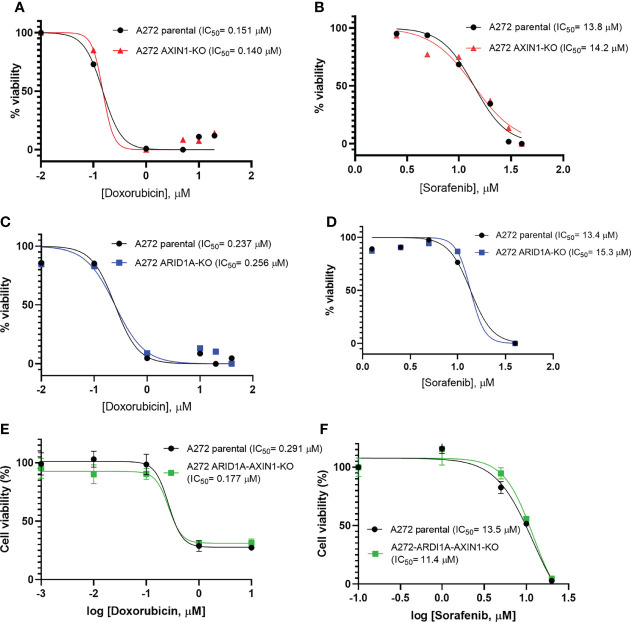
Knockout of *ARID1A* and/or *AXIN1* does not alter the susceptibility of porcine HCC cells to sorafenib nor doxorubicin. A272 porcine HCC cells were incubated with serial dilutions of doxorubicin or sorafenib and viability was measured after 48 hours. Relative cell viability is plotted against log concentration of doxorubicin **(A, C, E)** or sorafenib **(B, D, F)**. No significant difference was detected in log half-maximal inhibitory concentration (IC_50_) of parental A272 cells as compared to *AXIN1* ^KO^ **(A, B)**, *ARID1A* ^KO^ **(C, D)**, or *ARID1A-AXIN1* ^KO^ **(E, F)** cells at p < 0.05. IC_50_ values are shown in the figures.

Doxorubicin is the most common cytotoxic agent used as a monotherapy in transarterial chemoembolization, which is standard treatment for intermediate stage HCC (4, 26). Sorafenib was the only approved systemic therapy for nearly a decade, and is still widely used as a first line treatment, for advanced stage HCC (3, 27). Due to their widespread use in intermediate and advanced stage HCC, the effect of *AXIN1* and/or *ARID1A* KO on the susceptibility of porcine HCC cells to doxorubicin and sorafenib was investigated. A272 *AXIN1* ^KO^, *ARID1A* ^KO^, and *ARID1A-AXIN1* ^KO^ cells did not exhibit differences in doxorubicin or sorafenib IC_50_ as compared with parental cells (**Figure 7**).

### Development of Genetically-Tailored Subcutaneous Tumors in Oncopigs

**Figure 8:**
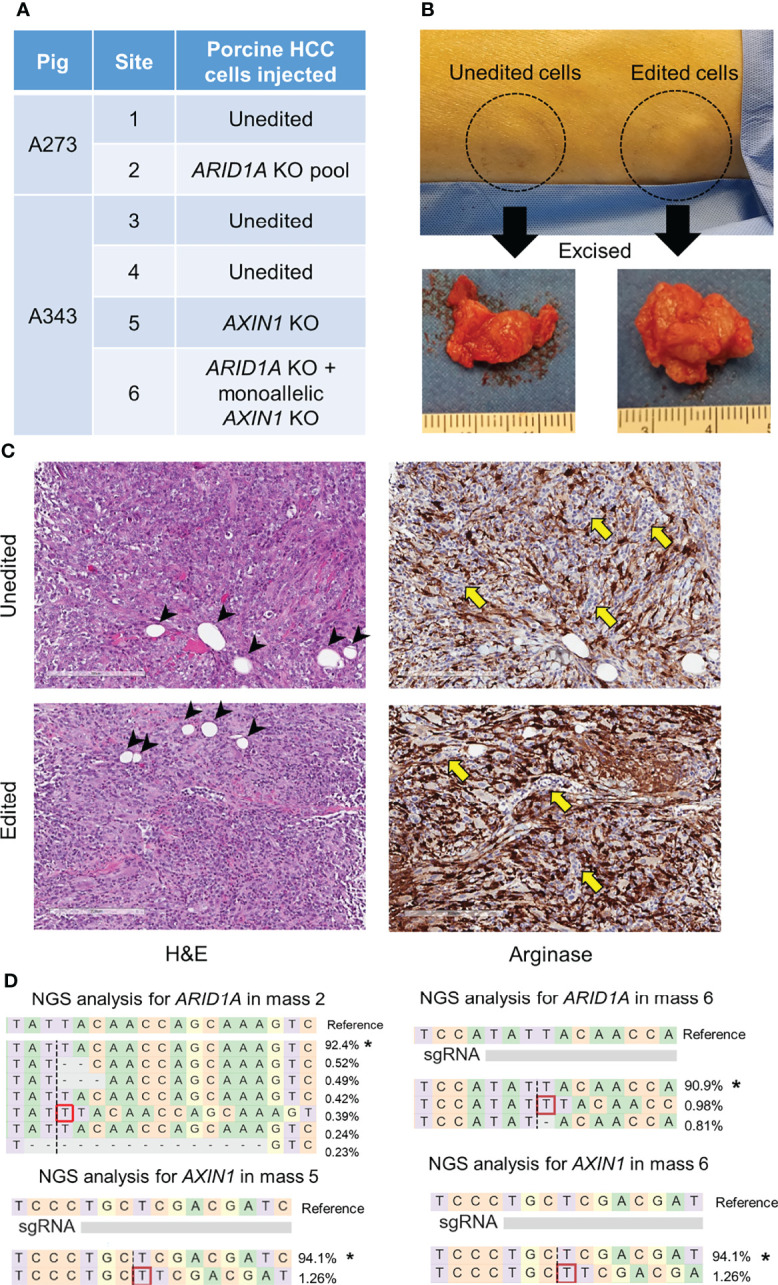
Development of subcutaneous masses harboring CRISPR-induced *ARID1A* and/or *AXIN1* edits in pigs. **(A)** Autologous unedited or CRISPR-edited porcine HCC cells were injected into distinct subcutaneous sites in 2 Oncopigs as presented in the table. **(B)** Representative image of two subcutaneous masses developed in pig flank 11 days post-injection of unedited or CRISPR-edited porcine HCC cells, at which point the masses were excised. **(C)** Representative microscopy images of H&E and Arginase staining of subcutaneous masses developed by injection of unedited or CRISPR-edited cells. The images show Arginase-stained porcine HCC cells (brown staining) surrounded by inflammatory cells (yellow arrows) and fat cells (arrow heads). Scale bar, 200 μm. **(D)** Reads detected by targeted NGS analysis in the three CRISPR-edited subcutaneous masses mapped to the reference sequences. The percentage of reads of each sequence are shown on the right. The asterisk (*) indicates/marks non-edited reads. Dashed line, predicted Cas9 cleavage position; red box, insertion; dash, deleted base.

CRISPR-edited porcine HCC cells were injected subcutaneously in pigs resulting in mass formation within a week (**Figures 8**, **S1**, **S2**), consistent with previous studies using unedited porcine HCC cells (17, 21). A273 porcine HCC cell pool with 98.5% *ARID1A* disruptions was autologously injected into a subcutaneous site in Oncopig A273 (**Figures 8A**, **S1**). A273 unedited porcine HCC cells were injected into an adjacent subcutaneous site in Oncopig A273 as a control (**Figure 8A**). Oncopig A343 received 4 subcutaneous injections of autologous HCC cells. Unedited A343 porcine HCC cells were injected in two sites as controls. A343 *AXIN1* ^KO^ porcine HCC clonal line was injected in a site and A343 porcine HCC clonal line with *ARID1A* ^KO^ and monoallelic KO of *AXIN1* was injected into another subcutaneous site (**Figure S1**). All these injections resulted in development of subcutaneous tumors (**Figures 8B**, **S2**). Histological analysis of these masses showed Arginase-stained cells confirming the presence of the porcine HCC cells in the subcutaneous mass formed (**Figure 8C**). The Arginase-stained cells recapitulated liver morphology, and were interspersed with collagen. Histological analysis also showed the presence of fat cells typically seen in subcutaneous tissues, inflammatory infiltration, and blood vessels (**Figure 8C**). NGS analysis confirmed the expected edits in *ARID1A* and *AXIN1* in the 3 masses resulting from injection of CRISPR-edited cells (**Figure 8D**). These results demonstrate the feasibility of development of genetically tailored tumors by autologous injection in pigs.

## Discussion

The ability to precisely modify animal genes is highly valuable for functional investigation of disease-related mutations and for generation of models that faithfully recapitulate human diseases. In this study, we employed CRISPR/Cas9 to knockout *AXIN1* and/or *ARID1A* in porcine HCC cells and investigated the functional effects of these HCC-related genetic alterations. Pigs share many similarities with humans, including similarities in anatomy, immunity, genetics, and metabolism (28). This increases the rate of successful translation of studies done using porcine models into clinical success and promotes the use of porcine models as a bridge between murine studies and clinical practice. Furthermore, the similarity in size between pigs and humans allows testing devices and procedures not feasible to test in small animal models such as mice. Size similarity is particularly important in HCC models due to the widespread use of image-guided intra-arterial therapies in clinical practice (4). Together, these factors make porcine models valuable tools for translational HCC studies. With recent development of an orthotopic implantation porcine HCC model and advances in CRISPR/Cas9 gene editing, it is possible to develop genetically-tailored HCC tumors in pigs by implantation of autologous CRISPR-edited HCC cells. The availability of such precision large animal models can accelerate the development and testing of precision medicine approaches, leading to improvement of the outcome of HCC patients.

*AXIN1* is one of the most frequently mutated genes in human HCC (9, 10, 29). The majority of *AXIN1* mutations in HCC are loss-of-function mutations, due to an early stop codon or splice site mutation, combined with loss of heterozygosity (30). In this study, we designed and validated a CRISPR gRNA that successfully disrupts porcine *AXIN1* gene. Among the three tested gRNAs, the gRNA that resulted in the highest editing efficiency had an “A” nucleotide at position -4 from the protospacer adjacent motif (PAM) sequence, and mostly resulted in a +1 insertion, whereas the other two gRNAs which were less efficient, had a “G” nucleotide at this position and mostly resulted in small deletions. These editing results are consistent with the work of Chakrabarti and colleagues, who analyzed indels at over 1,000 genomic sites in human cells and demonstrated that CRISPR editing outcomes can be predicted based on factors including the fourth nucleotide upstream of the PAM (31). Their work showed that majority of target sites that contained an “A” or a “T” at position -4 from the PAM exhibited higher editing precision and efficiency, and were most frequently repaired by insertion of A or T, respectively. In contrast, most targets containing a ‘‘G’’ at position -4 showed deletions and had lower editing efficiency. Indeed, gRNA#2 resulted in consistent editing outcome across all the porcine HCC cells used in this study. Using this gRNA, pure genetically-validated *AXIN1* ^KO^ clonal populations were isolated from porcine HCC cells, and were used for functional assays.

The effects of hepatocyte-specific deletion of *AXIN1* in mice have been described in two reports. In both the studies, liver specific AXIN1 loss resulted in HCC development with a low penetrance and after a significant latency period (30, 32). This suggests that *AXIN1* loss-of-function alone is only mildly oncogenic and likely requires additional oncogenic events that may differ between tumors and hence contribute to human HCC heterogeneity (30). In the current study, *AXIN1* KO increased the migration but did not alter the proliferation of porcine HCC cells. This is in line with a recent study where knockdown of *AXIN1* did not alter the growth of porcine inducible pluripotent stem cells (iPSCs) (33) and another study where restoring full−length *AXIN1* expression did not alter proliferation of SNU449 human HCC cells (34). However, these results are inconsistent with two studies where overexpression of *AXIN1* inhibited growth of SNU475 and SNU423 human hepatoma cell lines and SK-HEP-1 human hepatic adenocarcinoma cell line (35, 36). These differences could be stemming from alterations in the signaling pathways in these different cell lines.

*ARID1A* is an epigenetic regulator that is mutated in nearly 6% of cancers, including HCC, ovarian clear cell cancers, uterine endometrioid cancers, and gastric cancers. In HCC, negative *ARID1A* expression was significantly associated with larger tumor size, metastasis, shorter recurrence-free survival, and shorter overall survival (37, 38). Similar to *AXIN1*, liver specific deletion of *ARID1A* alone could not initiate liver cancer in mice (39), albeit it enhanced diethylnitrosamine (DEN)-induced hepatocarcinogenesis (40). Interestingly, the work of Sun and colleagues demonstrated that the role of *ARID1A* in HCC is context-dependent (41). While liver-specific deletion of *ARID1A* in mice conferred resistance to tumor initiation, its deletion in established tumors facilitated tumor progression and metastasis. This indicated that *ARID1A* functions in an oncogenic capacity during tumor initiation and a tumor suppressor capacity during tumor progression and metastasis (41). Consistently, *ARID1A* was found to be highly expressed in some human primary HCC tumors but not in metastatic lesions, suggesting that it can be lost after initiation (41).

In the current study, *ARID1A* KO increased porcine HCC cell proliferation and migration. This is consistent with findings of other studies that used human HCC cell lines. In the study by He and colleagues, *ARID1A* knockdown increased cell migration and invasion *in vitro* in MHCC-97H and Huh7 HCC cell lines and increased tumor growth *in vivo* in a xenografted HCC tumor model (37). Similarly, knockdown of *ARID1A* increased proliferation and migration in MHCC-97H and MHCC-97L cells (42) and *ARID1A* KO increased proliferation of Bel7404 cells (43).

Employing multiplexed CRISPR editing can expand the mutational profiles represented by precision porcine HCC models and enable modeling tumor heterogeneity. This was successfully achieved *in vitro* in the current study, wherein CRISPR-mediated simultaneous disruption of *AXIN1* and *ARID1A* was performed in porcine HCC cells. A clonal population with KO of both *AXIN1* and *ARID1A* was isolated, and found to exhibit increased migration compared to parental cells. Interestingly, these cells proliferated at a similar rate with parental cells, similar to cells with KO of *AXIN1* alone, but in contrast to the effect of *ARID1A* KO. Elucidating the downstream signaling pathways in cells with KO of *AXIN1* and/or *ARID1A* would be necessary to define the underlying molecular mechanisms regulating their effect on cancer cell proliferation and migration.

Identifying therapeutic vulnerabilities based on tumor genetic alterations paves the path for patient stratification to more effective precision therapeutics. In this study, KO of *AXIN1* and/or *ARID1A* did not alter the response of porcine HCC cells to doxorubicin or sorafenib. A recent study showed that *ARID1A* deficiency promotes angiogenesis leading to HCC progression. Further, *ARID1A* loss sensitized tumors to the anti-angiogenic agent sorafenib *in vivo* (44). In line with our findings, knockdown of *ARID1A* in HCC cells did not alter susceptibility to sorafenib *in vitro*, implying that the increased susceptibility to sorafenib is not a direct effect of *ARID1A* loss in the HCC cells, rather it is due to the effect on angiogenesis. Although numerous studies have identified therapeutic vulnerabilities caused by *ARID1A* loss in ovarian clear cell carcinoma, including sensitivity to EZH2 inhibitors, Histone deacetylases (HDAC) inhibitors, and glutathione inhibitors (45–48), little is known about therapeutic selection conferred by *ARID1A* or *AXIN1* loss in HCC. Based on enrichment of human and mouse *AXIN1*-mutated HCCs in Notch and YAP oncogenic signatures (30), inhibition of these pathways is a potentially promising therapeutic strategy. Further mechanistic studies are needed to identify molecularly targeted therapies that exploit the consequences of these frequently occurring mutations.

As proof of concept for the development of genetically-tailored tumors in pigs, autologous injection of cells with *AXIN1* and/or *ARID1A* disruption resulted in the formation of subcutaneous masses harboring the expected CRISPR edits. These injections included a heterogeneous cell pool with *ARID1A* editing, a clonal *AXIN1* KO population, and a clonal population with complete KO of *ARID1A* and monoallelic KO of *AXIN1*. These results lay the foundation for developing intrahepatic genetically-edited tumors that allow testing precision medicine, diagnostics, and imaging approaches.

One of the limitations of this study is the low percentage of edited cells in masses. This could be due to the presence of other cell types in the analyzed samples, including inflammatory and fat cells. Due to heterogenous sampling bias, some samples could contain a larger tumor fraction than the others. Another limitation of this study was the small number of developed subcutaneous tumors, which did not allow comparing growth rates of CRISPR edited cells with unedited control cells.

To conclude, this study elucidates the effects of CRISPR KO of *AXIN1* and/or *ARID1A* on porcine HCC cell proliferation, migration, and therapeutic susceptibility to sorafenib and doxorubicin. Further, the study demonstrates feasibility of development of genetically tailored tumors in pigs by autologous cell injections. This leads the path to expanding the currently available Oncopig HCC model to additional genetically tailored models by intrahepatic implantation of CRISPR edited cells. Optimization of *in vivo* delivery of CRISPR components to hepatocytes would enable the development of precision porcine HCC models by *in vivo* CRISPR editing of HCC driver genes. These translational HCC models are promising tools for testing innovative precision medicine delivered by systemic and/or locoregional routes and matching frequently occurring gene mutations with effective therapeutics.

## Data Availability Statement

The raw data supporting the conclusions of this article will be made available by the authors, without undue reservation.

## Ethics Statement

The animal study was reviewed and approved by The University of Illinois Institutional Animal Care and Use Committee (IACUC).

## Author Contributions

RG, KS, and LS conceived the study. LE, KD, WT, MC, and MR performed the experiments. GG performed histological analysis. LE, KD, and MR conducted data analysis. LE designed the experiments and drafted the manuscript. RG and KS received funding. All the authors revised and approved the manuscript.

## Funding

This work was supported by the National Institutes of Health – National Cancer Institute (1R21CA219461) and the Department of Radiology, University of Illinois at Chicago.

## Conflict of Interest

LS, RG, and KS have received research support from the United States Department of Defense, the United States National Institutes of Health, Guerbet USA LLC, Janssen Research & Development LLC, NeoTherma Oncology, and TriSalus Life Sciences, and are scientific consultants for Sus Clinicals Inc.

The remaining authors declare that the research was conducted in the absence of any commercial or financial relationships that could be construed as a potential conflict of interest.

## Publisher’s Note

All claims expressed in this article are solely those of the authors and do not necessarily represent those of their affiliated organizations, or those of the publisher, the editors and the reviewers. Any product that may be evaluated in this article, or claim that may be made by its manufacturer, is not guaranteed or endorsed by the publisher.

## Supplementary Material

Supplementary Figure 1 NGS analysis of CRISPR-edited cells injected autologously in subcutaneous sites in the pigs.

Supplementary Figure 2 Histological images of subcutaneous masses developed in pigs. **(A)** Low magnification microscopy images of H&E stained subcutaneous masses developed in Oncopig A273. These masses were extracted 11 days after injection of autologous cells. Scale bar, 300 μm. **(B)** Low magnification microscopy images of H&E and Arginase stained subcutaneous masses developed in Oncopig A343. These samples were collected by an ultrasound-guided biopsy of the masses 8 days after cell injection. The black rectangles mark the areas magnified and presented in **Figure 8**. Scale bar, 300 μm.
