# Supplementary figures and images for: Effect of CRISPR Knockout of AXIN1 or ARID1A on Proliferation and Migration of Porcine Hepatocellular Carcinoma
Source: Front Oncol. 2022 May 20;12:904031. doi: 10.3389/fonc.2022.904031 (PMC9163418; doi:10.3389/fonc.2022.904031)

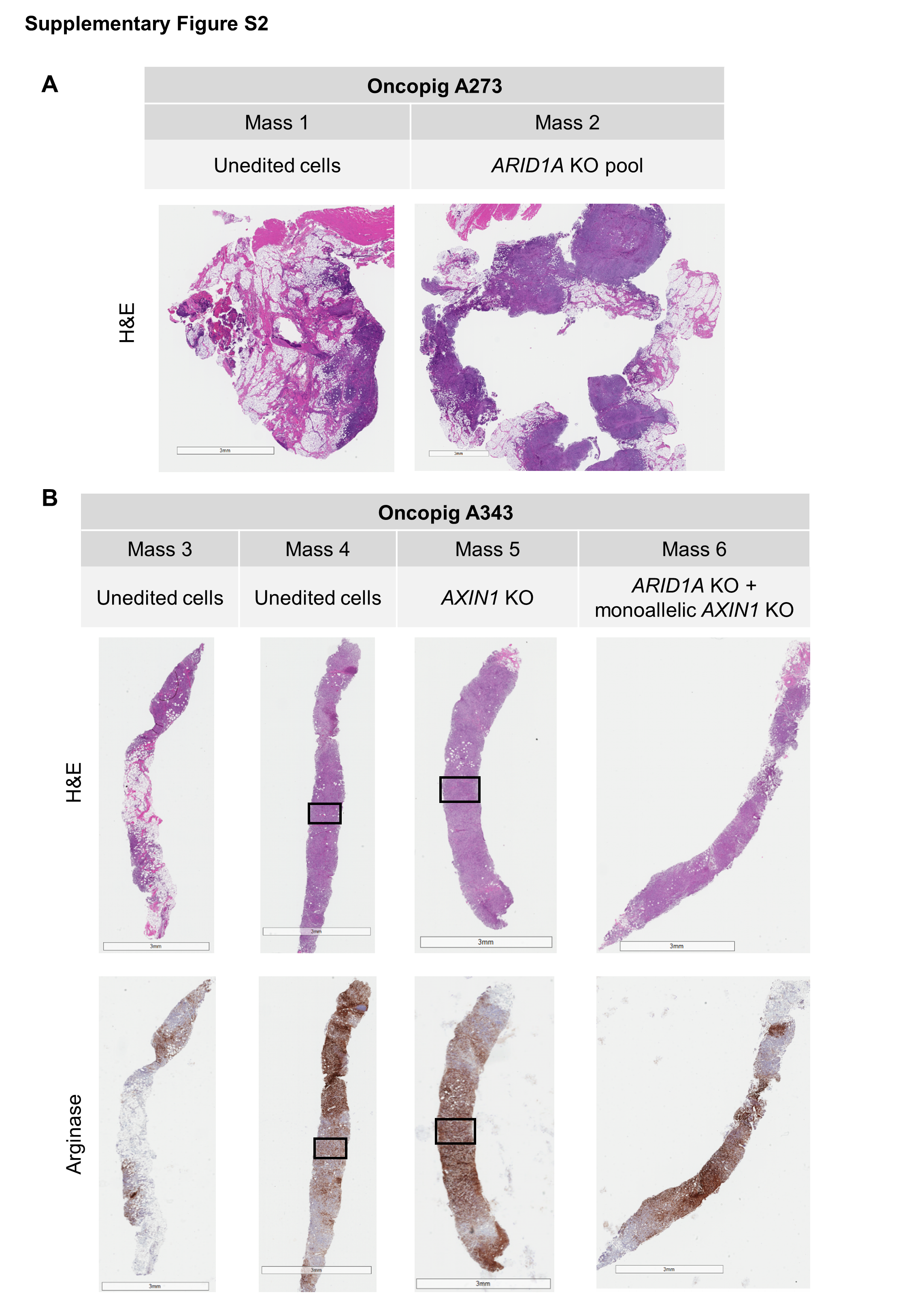

Supplement: Supplementary Figure 1 — NGS analysis of CRISPR-edited cells injected autologously in subcutaneous sites in the pigs. [file Image_1.tif]

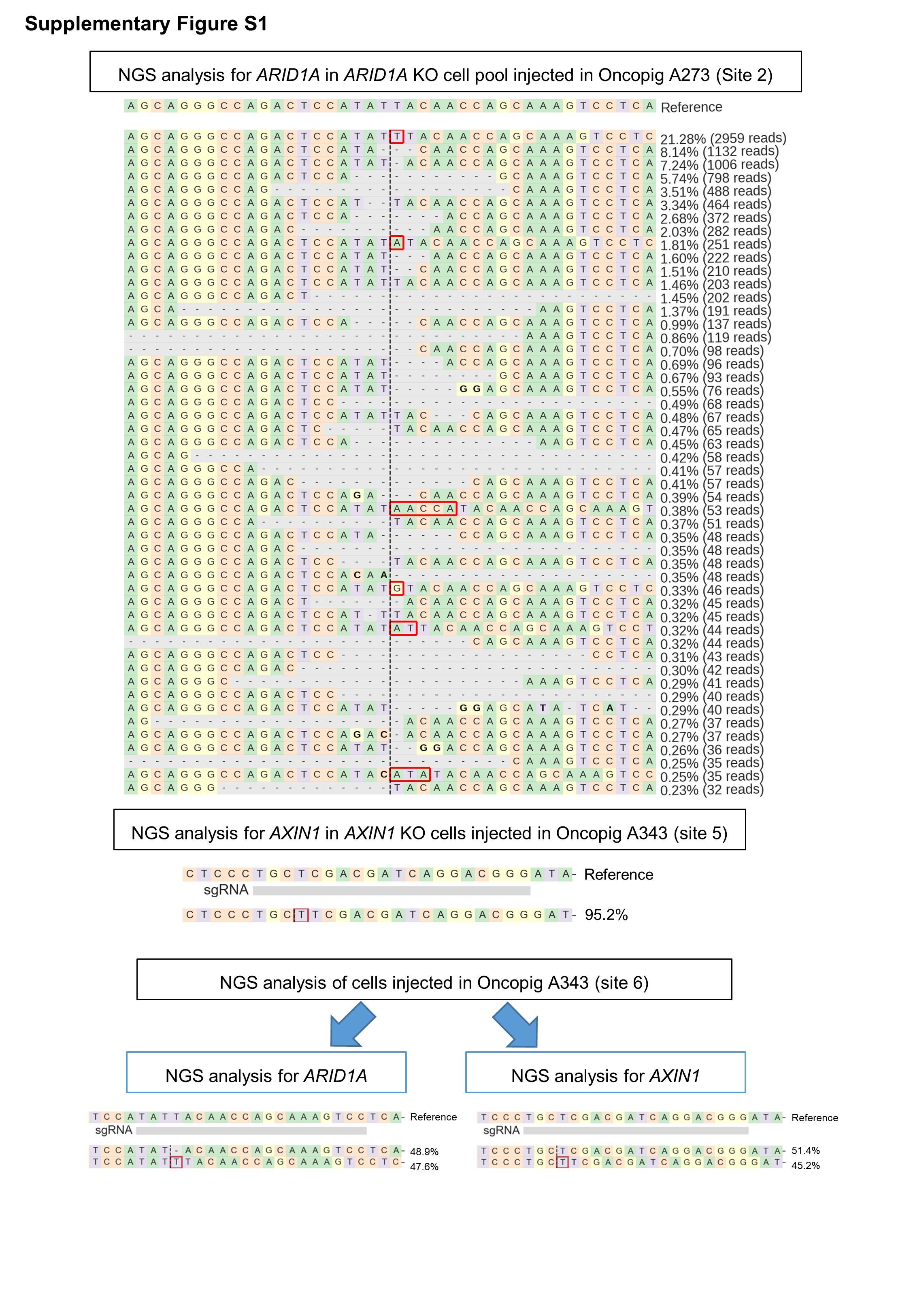

Supplement: Supplementary Figure 2 — Histological images of subcutaneous masses developed in pigs. (A) Low magnification microscopy images of H&E stained subcutaneous masses developed in Oncopig A273. These masses were extracted 11 days after injection of autologous cells. Scale bar, 300 μm. (B) Low magnification microscopy images of H&E and Arginase stained subcutaneous masses developed in Oncopig A343. These samples were collected by an ultrasound-guided biopsy of the masses 8 days after cell injection. The black rectangles mark the areas magnified and presented in Figure 8 . Scale bar, 300 μm. [file Image_2.tif]
